# Transferability and Fine-Mapping of Genome-Wide Associated Loci for Adult Height across Human Populations

**DOI:** 10.1371/journal.pone.0008398

**Published:** 2009-12-22

**Authors:** Daniel Shriner, Adebowale Adeyemo, Norman P. Gerry, Alan Herbert, Guanjie Chen, Ayo Doumatey, Hanxia Huang, Jie Zhou, Michael F. Christman, Charles N. Rotimi

**Affiliations:** 1 Center for Research on Genomics and Global Health, National Human Genome Research Institute, National Institutes of Health, Bethesda, Maryland, United States of America; 2 Coriell Institute for Medical Research, Camden, New Jersey, United States of America; 3 Department of Genetics and Genomics, Boston University School of Medicine, Boston, Massachusetts, United States of America; Max Planck Institute for Evolutionary Anthropology, Germany

## Abstract

Human height is the prototypical polygenic quantitative trait. Recently, several genetic variants influencing adult height were identified, primarily in individuals of East Asian (Chinese Han or Korean) or European ancestry. Here, we examined 152 genetic variants representing 107 independent loci previously associated with adult height for transferability in a well-powered sample of 1,016 unrelated African Americans. When we tested just the reported variants originally identified as associated with adult height in individuals of East Asian or European ancestry, only 8.3% of these loci transferred (*p*-values≤0.05 under an additive genetic model with directionally consistent effects) to our African American sample. However, when we comprehensively evaluated all HapMap variants in linkage disequilibrium (*r* ^2^≥0.3) with the reported variants, the transferability rate increased to 54.1%. The transferability rate was 70.8% for associations originally reported as genome-wide significant and 38.0% for associations originally reported as suggestive. An additional 23 loci were significantly associated but failed to transfer because of directionally inconsistent effects. Six loci were associated with adult height in all three groups. Using differences in linkage disequilibrium patterns between HapMap CEU or CHB reference data and our African American sample, we fine-mapped these six loci, improving both the localization and the annotation of these transferable associations.

## Introduction

Human adult height (stature, MIM 606255) is a classic complex trait, influenced by many genes and environmental factors [1]. Twin, family, and adoption studies indicate that ∼80–90% of phenotypic variation in adult height in individuals of European ancestry is due to genetic variation [2]–[10]. For comparison, estimates of the amount of phenotypic variation in adult height explained by genetic variation are ∼40–60% for individuals of African ancestry [11]–[15] and ∼65% for individuals of Chinese ancestry [16]. These estimates clearly show that adult height is a highly heritable trait across human populations. However, these estimates provide no information as to whether the same genetic variants influence adult height across human populations.

Recent progress in dissecting the genetic architecture of adult height includes the identification of 106 common autosomal single nucleotide polymorphisms (SNPs) that were associated with stature in genome-wide association studies in populations of European ancestry [17]–[21]. Some of these associations were replicated in cohorts of African Americans [19], [21]. Taken together, this collection of genetic variants that underlie variation in adult height thus far explains only ∼5% of phenotypic variation [22]. Since these initial publications, an additional 46 common autosomal SNPs have been associated with adult height in individuals of Chinese Han [23], European [24], or Korean ancestry [25], for a total of 152 SNPs.

The importance of replication studies as part of the process of studying genome-wide association is well known and criteria for establishing positive replication have been suggested [26]. One criterion for replication is that the follow-up sample should be a random sample drawn from the same population as the discovery sample [26]. We distinguish replication from transferability on the basis that the latter applies if a follow-up sample is drawn from a different population than the discovery sample. Consequently, if an association replicates but does not transfer across populations, then the association is population-specific. In this study, we investigated the transferability of association with adult height for the 152 previously reported SNPs using a population-based sample of 1,016 unrelated African Americans enrolled from the Washington, DC metropolitan area in a genetic epidemiology project entitled the Howard University Family Study (HUFS). Our main objective was to assess transferability of reported genetic associations originally detected in individuals of East Asian or European ancestry to our sample of African Americans. For those associations that transferred across all three groups, we used differences in linkage disequilibrium patterns across human populations to localize the loci, thereby allowing for improved annotation.

## Results

**Figure 1:**
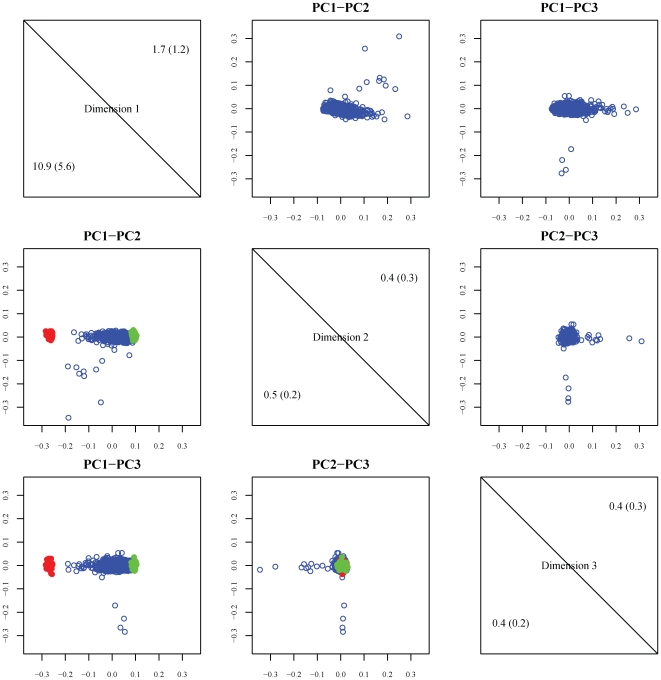
Principal coordinate analysis. Shown are the first three dimensions from classical multidimensional scaling of the allele sharing distance matrix. Red represents individuals from the HapMap CEU sample, green represents individuals from the HapMap YRI sample, and blue represents individuals from the HUFS sample (African Americans). The lower panels show two-dimensional projections of the first three dimensions for the HUFS sample including the CEU and YRI reference samples. The upper panels show two-dimensional projections of the first three dimensions for just the HUFS sample. The diagonal panels show the eigenvalues and in parentheses the variance explained by the first three dimensions.

From the entire HUFS sample of 1,976 individuals, we extracted a subset of 1,055 unrelated individuals. We identified 37 individuals as outliers, whereas the remaining 1,018 individuals formed one cluster (Fig. 1). Based on STRUCTURE analysis, the estimated individual proportion of African ancestry was 0.782±0.110. The projection of the first two principal coordinates from multi-dimensional scaling analysis including representative founder samples also shows the presence of admixture (Fig. 1). The variance inflation factor for genomic control was estimated to be 1.03 (Supplementary Fig. S1), indicating that the test statistics genome-wide were not substantially inflated and that residual population stratification was not a concern.

We investigated whether heritability of adult height among African Americans in the HUFS is more similar to heritability among African individuals or to heritability among European individuals. For the HUFS sample, the estimated heritability of adult height was 0.697 (SE 0.006) in a sample of 1,006 African American individuals in 326 families, intermediate between heritability estimates for adult height in African and European individuals. We hypothesize that this estimate is larger than previous estimates for African individuals because of a more similar environment between Europeans and African Americans than between African Americans and Africans and because of admixture. It is critical to note that similar heritability estimates do not imply that the same genetic variants influence adult height in these different samples.

**Figure 2:**
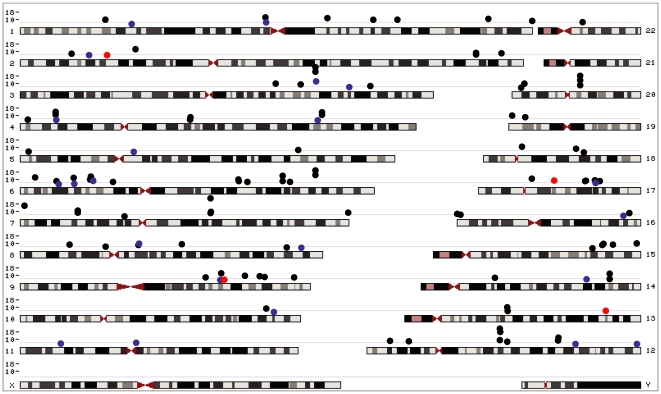
Genomic locations of SNPs previously associated with adult height. Black dots indicate associations originally discovered in populations of European ancestry. Red dots indicate associations originally discovered in populations of Chinese Han ancestry. Blue dots indicate associations originally discovered in populations of Korean ancestry. The y-axis represents discovery *p*-values on the −log_10_ scale. The light gray line indicates a *p*-value of 5×10^−7^.

To assess the transferability of genetic associations previously identified for adult height in populations of East Asian or European ancestry to African Americans, we collated 77 autosomal SNPs strongly associated (reported *p*-values≤5×10^−7^) with human height under an additive model and 75 autosomal SNPs suggestively associated (reported *p*-values ranging from 4.5×10^−3^ to 5×10^−7^) with human height under an additive model (Fig. 2) [17]–[21], [23]–[25]. Before testing transferability, we performed a power analysis. Based on the HUFS sample size of 1,016 unrelated individuals (Table 1), we estimated 80% power at a significance level of 0.05 to detect effect sizes of 0.12 cm and 0.03 cm under an additive model at a minimum minor allele frequency of 0.01 and an average minor allele frequency of 0.22, respectively. These estimates indicate that our sample was well-powered given previously reported effect sizes between 0.2 cm and 0.6 cm.

**Table 1:** Summary of the Howard University Family Study unrelated participants.

Characteristic	Male	Female
Sample size	419	597
Age (years)	47.9 (12.4)^a^	48.7 (13.7)
Height (cm)	175.7 (7.5)	162.9 (7.4)

a Shown are means (standard deviations).

**Figure 3:**
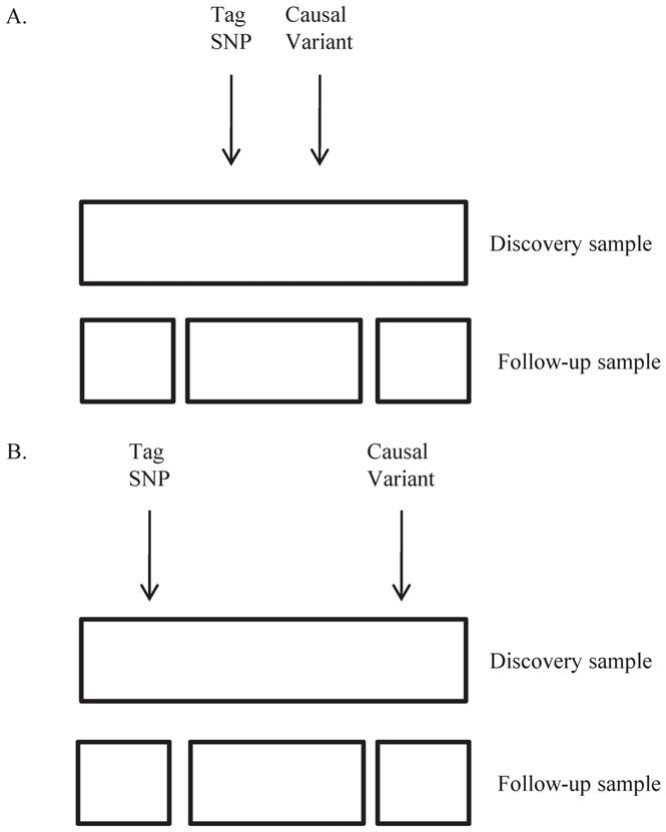
Schematic diagram of different linkage disequilibrium patterns in discovery and follow-up samples. A) The associated tag SNP and a causal variant are in the same region of linkage disequilibrium in both the discovery and follow-up samples. Both the exact and local approaches may yield successful transfer. B) The associated tag SNP and a causal variant are not in the same region of linkage disequilibrium in both the discovery and follow-up samples. The exact approach will fail for the original tag SNP but the local approach may succeed for other proxy SNPs if they are in the same region of linkage disequilibrium as the causal variant in the follow-up sample.

We took two approaches to evaluating transferability. First, we directly evaluated the previously reported SNPs using what has been referred to as an “exact” approach [27]. The power of this approach relies on the assumption that the previously associated marker and the causal variant(s) remain in linkage disequilibrium across populations (Fig. 3A). By accounting for linkage disequilibrium (*r* ^2^≥0.3) between SNPs in the HapMap CEU and CHB samples, we determined that the 152 SNPs represent 107 independent loci. Using this approach, we detected significant transfer to our African American sample for 7 of 84 (8.3%) testable loci (Supplementary Table S1).

Second, we comprehensively evaluated all HapMap Phase II SNPs in the region of linkage disequilibrium (*r* ^2^≥0.3) surrounding the previously reported SNPs using either CEU (for SNPs reported in studies of European-ancestry samples) or CHB (for SNPs reported in studies of East Asian-ancestry populations) data as appropriate, an approach referred to as “local” [27]. The power of this approach relies on the assumption that the previously associated marker points to a region of linkage disequilibrium in the discovery sample and that any SNP in such a region is potentially a proxy SNP for the causal variant(s) (Fig. 3B). Using this approach, we detected significant transfer to our African American sample for 53 of 98 (54.1%) testable loci (Supplementary Table S1). Taken together, these findings suggest that previously associated SNPs and causal variants are often not in linkage disequilibrium in our African American sample although they are in linkage disequilibrium in the discovery samples.

The transferability rate was 70.8% for the 77 variants that originally showed strong associations (*p*-values≤5×10^−7^) and 38.0% for the 75 variants that originally showed suggestive associations (*p*-values ranging from 4.5×10^−3^ to 5×10^−7^; Supplementary Table S1). The discovery *p*-values for associations that transferred to our African American sample ranged from 2.7×10^−3^ to 1.4×10^−27^ (Supplementary Table S1). These findings support the hypothesis that genuine associations exist with *p*-values not meeting strict genome-wide significance levels. Encouragingly, all associations with discovery *p*-values≤10^−13^ transferred to our African American sample (Supplementary Table S1).

An important factor that is likely to influence the rate of transferability in our study is coverage of genetic variation. We examined this issue by estimating how well our admixed African American sample consisting of ∼2.4 million experimentally determined and imputed genotyped SNPs covered the variation in the HapMap CEU and CHB samples. Our calculations show that coverage is 71.2% for HapMap CEU variation and 75.8% for HapMap CHB variation (Supplementary Table S1). Due to this limitation, it is possible that we underestimated transferability.

### Fine-Mapping

For associations discovered in populations with longer-range linkage disequilibrium patterns, follow-up in a population with shorter-range linkage disequilibrium patterns offers the opportunity for *in silico* fine-mapping [28]. Thus, we investigated whether the African American sample provided refined localization for the six loci that transferred across all three population groups (African Americans, East Asians, and Europeans).

**Figure 4:**
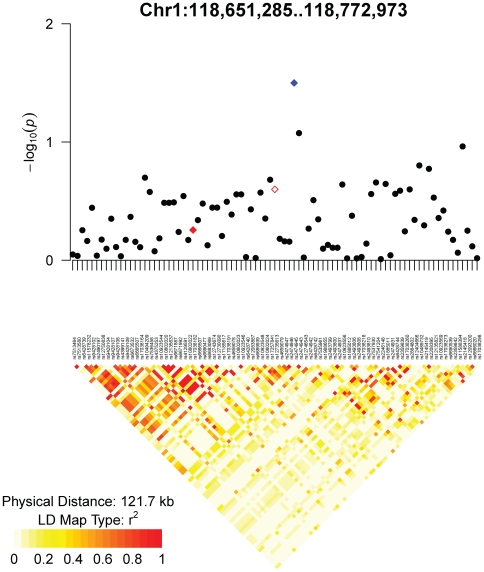
Association *p*-values and linkage disequilibrium in the HUFS sample for the height locus at chromosome 1p12. The open red diamond indicates SNP rs12735613 (for which the association was discovered in individuals of European ancestry), the filled red diamond indicates rs17038182 (for which the association was discovered in individuals of Korean ancestry), and the blue diamond indicates the SNP associated in the HUFS (African American) sample. The boundaries of the set of SNPs reflect *r* ^2^≥3 in the HapMap CHB data surrounding rs17038182.

The association of SNP rs12735613 at 118,685,496 bp on chromosome 1 was originally discovered in individuals of European ancestry [20]. The association of proxy SNP rs17038182 at 118,669,928 bp was discovered in individuals of Korean ancestry [25]. We found that rs2474945 at 118,686,437 bp was the only SNP in the region of linkage disequilibrium surrounding rs12735613 or rs17038182 for which the association transferred to our sample of African American individuals (Fig. 4 and Supplementary Table S1). rs2474945 is 157 kb upstream of the gene *SPAG17* (GeneID 200162). Importantly, neither rs12735613 nor rs17038182 themselves are significantly associated with adult height in our sample (Supplementary Table S1). Thus, this locus exemplifies the situation depicted in Fig. 3B, in which the exact approach fails to yield significant transfer but the local approach succeeds.

The association of rs3791679 at 55,950,306 bp on chromosome 2 was originally discovered in individuals of European ancestry [21]. The proxy SNP rs3791675 at 55,964,813 bp was found to transfer in individuals of Korean ancestry [25]. We found that the association at both rs3791679 and rs3791675 transferred to our sample of African American individuals, as well as the association at rs7571341 at 55,924,566 bp (Supplementary Table S1). SNPs rs3791679 and rs3791675 are intronic in and rs7571341 is 22 kb downstream of the gene *EFEMP1* (GeneID 2202).

The associations of rs6440003 at 142,576,899 bp on chromosome 3 [20], rs6763931 at 142,585,523 bp [21], and rs724016 at 142,588,260 bp [18] were all originally discovered in individuals of European ancestry. The association of rs1051317 at 142,626,120 bp was discovered in individuals of Korean ancestry [25]. We found six SNPs (rs9829470, rs9821337, rs9822195, rs13091182, rs6785073, and rs6789653) from 142,536,380 bp to 142,633,680 bp for which the association transferred to our sample of African American individuals (Supplementary Table S1). SNPs rs9829470, rs9821337, and rs9822195 are all upstream of the gene *ZBTB38* (GeneID 253461) and SNPs rs13091182, rs6785073, and rs6789653 are all intronic in the same gene.

The associations of rs6842303 at 17,463,153 bp on chromosome 4 [21], rs16896068 at 17,553,938 bp [20], and rs6830062 at 17,626,828 bp [21] were all originally discovered in individuals of European ancestry. The association of rs2011603 at 17,643,582 bp was discovered in individuals of Korean ancestry [25]. We found six SNPs (rs16895878, rs16895895, rs16895971, rs13141926, rs13103931, and rs2707450) from 17,456,379 bp to 17,551,658 bp for which the association transferred to our sample of African American individuals (Supplementary Table S1). SNP rs16895878 is in the 3′ UTR of and rs16895895, rs16895971, rs13141926, rs13103931, and rs2707450 are intronic in the gene *LCORL* (GeneID 254251).

The associations of rs10958476 at 57,258,362 bp at chromosome 8 [21] and rs9650315 at 57,318,152 bp [18] were originally discovered in individuals of European ancestry. The association of rs13273123 at 57,263,345 bp was discovered in individuals of Korean ancestry [25]. We found nine SNPs (rs6987156, rs6474053, rs7829319, rs7815788, rs13272414, rs4469431, rs13248165, rs13275320, and rs7460090) from 57,332,019 bp to 57,356,717 bp that transferred to our sample of African American individuals (Supplementary Table S1). These SNPs are 38–63 kb downstream of the gene *CHCHD7* (GeneID 79145), 18–43 kb downstream of the gene *RDHE2* (GeneID 195814), and 46–70 kb upstream of the gene *PLAG1* (GeneID 5324).

**Figure 5:**
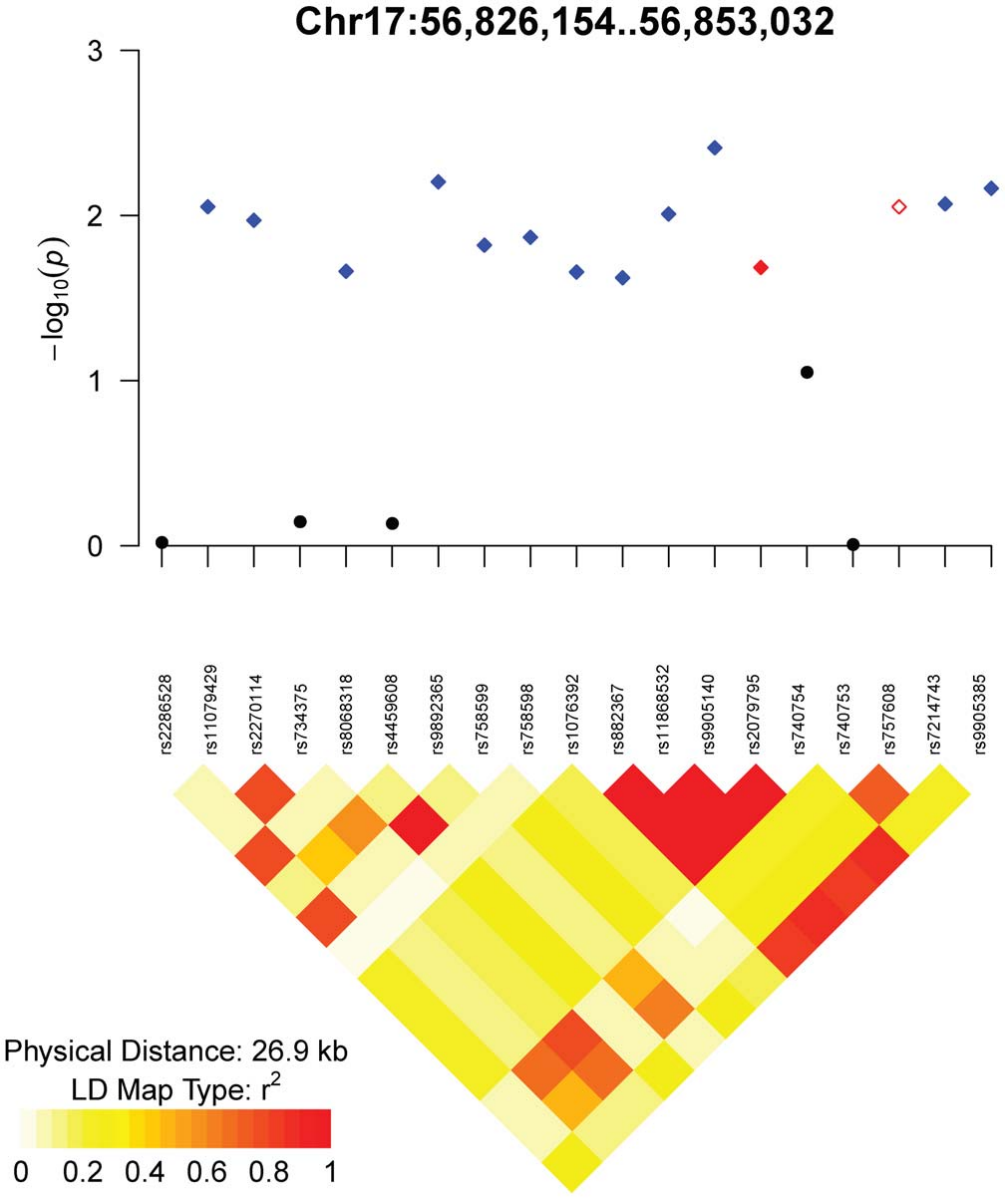
Association *p*-values and linkage disequilibrium in the HUFS sample for the height locus at chromosome 17q23.2. The open red diamond indicates SNP rs757608 (for which the association was discovered in individuals of European ancestry), the filled red diamond indicates rs2079795 (for which the association was discovered in individuals of Korean ancestry), and blue diamonds indicate SNPs associated in the HUFS (African American) sample. The boundaries of the set of SNPs reflect *r* ^2^≥3 in the HapMap CHB data surrounding rs2079795.

The association of rs757608 at 56,852,059 bp on chromosome 17 [21] was originally discovered in individuals of European ancestry. The association of rs2079795 at 56,851,431 bp was discovered in individuals of Korean ancestry [25]. In addition to these two SNPs, we found 12 SNPs (rs11079429, rs2270114, rs8068318, rs9892365, rs758599, rs758598, rs1076392, rs882367, rs11868532, rs9905140, rs7214743, and rs9905385) from 56,827,185 bp to 56,853,032 bp for which the association transferred to our sample of African American individuals (Fig. 5 and Supplementary Table S1). These SNPs are 14 kb upstream through 11 kb downstream of the gene *TBX2* (GeneID 6909) and 36–61 kb upstream of the gene *TBX4* (GeneID 9496). This locus exemplifies the situation depicted in Fig. 3A, in which the exact approach yields significant transfer.

To investigate whether these 14 SNPs reflect one underlying signal, we tested each SNP in this region for association conditional on the SNP (rs9905140) with the strongest signal in the distal part of the region (Fig. 5). Conditioning on rs9905140 failed to completely eliminate the signal at the other SNPs (Table 2). Similarly, conditioning on rs9892365, the SNP with the strongest association in the proximal part of the region (Fig. 5), failed to completely eliminate the signal at the other SNPs (Table 2). However, conditioning on both rs9892365 and rs9905140 successfully eliminated the signal at the other SNPs (Table 2). These results suggest the presence of two associations within one region of linkage disequilibrium in the CEU and CHB samples but spanning multiple regions of linkage disequilibrium in our African American sample.

**Table 2:** Conditional analysis of the height locus at 17q23.2.

SNP	Position	unconditional *p* a	*p* conditional on rs9905140	*p* conditional on rs9892365	*p* conditional on rs9905140 and rs9892365
rs2286528	56,826,154	0.9553	0.3479	0.7710	0.6028
rs11079429	56,827,185	**0.0089**	0.2300	**0.0318**	0.2929
rs2270114	56,833,558	**0.0107**	0.2585	**0.0462**	0.3476
rs734375	56,836,155	0.7153	0.5489	0.4657	0.9817
rs8068318	56,838,548	**0.0218**	0.8584	0.1120	0.9261
rs4459608	56,845,812	0.7329	0.5953	0.4338	0.9686
rs9892365	56,846,166	**0.0063**	**0.0411**	NA	NA
rs758599	56,847,050	**0.0151**	0.4144	0.2222	0.8400
rs758598	56,847,496	**0.0136**	0.3571	0.2016	0.7555
rs1076392	56,847,790	**0.0220**	0.5024	0.2837	0.9688
rs882367	56,849,356	**0.0238**	0.5034	0.2518	0.9515
rs11868532	56,850,865	**0.0098**	0.6992	**0.0489**	0.7513
rs9905140	56,851,020	**0.0039**	NA	**0.0414**	NA
rs2079795	56,851,431	**0.0206**	0.3404	0.2483	0.7649
rs740754	56,851,642	0.0890	**0.0314**	0.1731	0.0707
rs740753	56,851,709	0.9833	0.3674	0.6103	0.8242
rs757608	56,852,059	**0.0089**	0.0653	0.4231	0.6324
rs7214743	56,852,834	**0.0085**	0.0584	0.4241	0.6359
rs9905385	56,853,032	**0.0069**	0.0575	0.4860	0.7215

a *P*-values less than 0.05 are shown in bold.

## Discussion

In this study, we sought to identify genetic variants influencing adult height in African Americans. Our study of 1,016 African Americans was well-powered to test for transferability of associations based on previously reported effect sizes. We found that 8.3% of genetic variants previously reported to influence adult height in individuals of East Asian or European ancestry also influence adult height in our sample of African Americans. However, when we comprehensively evaluated all HapMap SNPs in linkage disequilibrium with those genetic variants, we found that 54.1% of loci were associated with adult height in our sample of African Americans. Thus, it was uncommon for associations at tag SNPs on commercial chips to directly transfer across populations. Rather, it was more common that other proxy SNPs in linkage disequilibrium with the originally reported tag SNPs transferred across populations. Furthermore, six associated loci transferred across all three population groups. These observations argue strongly for the comprehensive evaluation of linkage disequilibrium as well as inclusion of populations with ancestries from different parts of the world as part of genome-wide association studies [26], [29], [30].

One of our criteria for declaring successful transfer was consistency in the direction of effect size estimates [26]. It has been noted that differences in haplotype frequencies and linkage disequilibrium, as well as other factors such as unmodeled interactions, can induce changes in the sign of effect size estimates [31], [32]. We therefore mention that 23 loci yielded significant *p*-values but directionally inconsistent effect size estimates.

Linkage disequilibrium has both positive and negative impacts on association testing. On the positive side, differences in linkage disequilibrium patterns can increase resolution for localizing indirect associations. Resolution generally increases as we fine-map a discovery in a sample of individuals of European ancestry using a follow-up sample of individuals of African ancestry due to the shorter range of linkage disequilibrium in the latter sample. We took advantage of the shorter range of linkage disequilibrium in our African American sample to localize the height loci originally reported in East Asians and Europeans. Our fine-mapping effort revealed evidence that one height locus at chromosome 17q23.2 appears to consist of two associations spanning multiple regions of linkage disequilibrium in our sample of African American individuals. Such a finding would not be possible if we had investigated just the reported SNPs (the exact approach). On the negative side, it is widely assumed that the sample size for testing indirect association scales inversely with the linkage disequilibrium *r^2^* between the typed marker and the untyped causal variant. However, this simple rule tends to underestimate the sample size necessary to maintain power to test indirect association [33].

A practical issue with the local approach is how to define the set of SNPs to be considered for replication or transferability. One possibility is to define the set based on the gene containing the original SNP, assuming that the original SNP is genic. Another possibility is to define the set based on linkage disequilibrium surrounding the original SNP, as we did in this study. If a gene spans multiple regions of linkage disequilibrium, then the latter choice is preferable because it requires less genotyping and induces a smaller statistical testing burden. The latter choice also applies whether or not the original SNP is genic.

In summary, we investigated genetic variants influencing adult height in African Americans. We found that 54.1% of loci previously associated with adult height in populations of East Asian or European ancestry transferred to our sample of African Americans. Our results highlight the importance of comprehensively evaluating all genetic variants in linkage disequilibrium with associated markers when testing for either replication or transferability. We successfully used the shorter range of linkage disequilibrium in our African American sample to refine the localization of the six height loci that transferred across African American, East Asian, and European samples.

## Materials and Methods

### Ethics Statement

Ethical approval for the Howard University Family Study (HUFS) was obtained from the Howard University Institutional Review Board and written informed consent was obtained from each participant.

### Study Samples

The HUFS is a population-based study of African American families enrolled from the Washington, D.C. metropolitan area. The main objectives of the HUFS are to: 1) enroll and examine a randomly ascertained cohort of 350 African American families with members in multiple generations from the Washington, D.C. metropolitan area; 2) characterize the study participants for anthropometry (including weight, height, waist and hip circumferences, and body composition measures), blood pressure (BP) and related physiologic intermediates, and diabetes-related and lipid-related variables; 3) evaluate the association between hypertension/blood pressure and selected candidate genes; and 4) store high-quality DNA to conduct studies to identify novel genomic regions linked and/or associated with common complex traits. Families were not ascertained based on any phenotype. In a second phase of recruitment, additional unrelated individuals from the same geographic area were enrolled to facilitate nested case-control study designs.

During a clinical examination, we collected demographic information and measured BP, anthropometry, and body composition (fat mass and fat-free mass). Blood was drawn for biochemical assays (sodium, potassium, creatinine, urea, and glucose) and several other molecular phenotypes (including cortisol, insulin, and leptin). The total number of recruited individuals was 2,028, of which 1,976 remained after data cleaning. From this sample, we extracted a subset of 1,055 unrelated individuals. The enrollment procedures (forms, measurements, and lab assays) for unrelated individuals were identical to those for the families. Height was measured with a stadiometer to the nearest 0.1 cm.

### Genotyping and Quality Control

Genome-wide genotyping was performed using the Affymetrix Genome-Wide Human SNP Array 6.0 and genotypes calls were made using the Birdseed algorithm, version 2 [34]. We had four genotype inclusion criteria: the individual sample success rate had to be ≥95% (no samples excluded), the SNP call rate had to be ≥95% (41,885 SNPs excluded), the minor allele frequency had to be ≥0.01 (19,154 SNPs excluded), and the *p*-value for the Hardy-Weinberg test of equilibrium had to be ≥1.0×10^−3^ (6,317 SNPs excluded). For the remaining 808,465 autosomal SNPs, the average call rate was 99.5%. The concordance of blind duplicates was 99.74%.

### Population Stratification

Evidence for population stratification was obtained through nonparametric clustering of genotypes using the R package AWclust [35]. Two-dimensional projections from principal coordinate analysis were drawn using R. From the set of 1,055 unrelated individuals, 37 individuals identified as outliers were removed from analysis. We used 10,000 random autosomal SNPs in linkage equilibrium for estimation of the allele sharing distance matrix. Two additional individuals were removed due to missing phenotype data, leaving 1,016 individuals for association analysis. We also estimated the variance inflation factor for genomic control.

### Admixture

Individual admixture proportions were estimated using a panel of 2,076 ancestry-informative markers (AIMs) assuming two populations and uncorrelated allele frequencies with a 10,000 step burn-in and a 1,000 step chain using STRUCTURE 2.2 [36]. AIMs had a minor allele frequency ≥0.01 in both the HapMap CEU and YRI samples, a difference in allele frequencies between the HapMap CEU and YRI samples ≥0.6, and a pairwise *r^2^*≤0.4 with other markers in the panel in both the HapMap CEU and YRI samples.

### Heritability

Heritability, with age and sex included as covariates, was estimated using SOLAR under a polygenic model [37]. The final sample for heritability estimation included 326 pedigrees, consisting of a total of 1,006 individuals.

### Imputation

Imputation was performed using MACH, version 1.0.16, available at http://www.sph.umich.edu/csg/abecasis/MACH/download/. We first retrieved the combined HapMap phase II+III raw genotype files from http://ftp.hapmap.org/genotypes/2009-02_phaseIIIII/forward/non-redundant/. We filtered the 3,907,239 autosomal CEU SNPs and the 3,860,794 autosomal YRI SNPs based on the inclusion of founders only, a minor allele frequency ≥0.01, a SNP missingness rate≤5%, and an individual missingness rate≤5%, leaving 2,327,370 CEU reference SNPs and 2,598,198 YRI reference SNPs. We inferred haplotype phases for the reference data using the settings–rounds 50–states 200. We conditioned imputation on the maximum-likelihood estimates of the crossover map, which specifies the likely locations of haplotype transitions, and the error rate map, which specifies unusual markers based on a combination of discrepancies between the reference panel and study sample data, genotyping error, and recurrent mutation. We calibrated imputation error by determining the threshold of posterior probability that yielded a 10% error rate for the CEU reference panel and a 5% error rate for the YRI reference panel, averaged over 6,800 SNPs for which we masked the experimentally determined genotypes. Imputed genotypes were passed through quality control filters of a minor allele frequency ≥0.01, a SNP missingness rate≤10%, and a Hardy-Weinberg test *p*-value ≥0.001. If a reference SNP yielded an imputed genotype for both the CEU and YRI reference panels, we preferentially accepted the genotype using the YRI reference panel. We successfully imputed 1,506,100 SNPs using the YRI reference panel and an additional 52,291 SNPs using the CEU reference panel, for a total of 2,366,856 experimentally determined and imputed autosomal SNPs (Supplementary Table S2). Quality control and data management were performed using PLINK, available at http://pngu.mgh.harvard.edu/purcell/plink/ [38].

### Linear Regression

We analyzed only height measurements for individuals at least 20 years old, *i.e.*, adult height. Height phenotypic measurements were approximately normalized using a log_10_ transformation. Normalized height was regressed on age, sex, and individual admixture proportion using R. Standardized residuals were regressed on genotype under the additive model using PLINK.

### Transferability

Transferability was assessed using the same criteria for replication: the same SNP has a significant association at 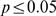 under the same genetic model with a consistent direction for the effect size estimate with respect to the HapMap reference allele [26]. We tested for transferability using two approaches. In the first approach, we directly tested just the originally reported SNP. In the second approach, we comprehensively evaluated all HapMap SNPs in linkage disequilibrium with the originally reported SNP. To accomplish this, we created a set containing all Phase II HapMap SNPs bounded by the farthest SNPs with pairwise 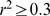 to the originally reported SNP in the CEU or CHB sample as appropriate. We determined marginal *p*-values for each SNP in the set. Using the union-intersection test for the set, the null hypothesis is that no single SNP within the set is significantly associated with adult height and the alternative hypothesis is that at least one single SNP within the set is significantly associated with adult height. Therefore, the *p*-value for the set equals the minimum of the marginal *p*-values for all SNPs in the set. We declared significance if the set 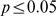. We did not correct for multiple comparisons across sets because the null hypothesis for each set is different and therefore the tests across sets do not constitute a family. Maps of linkage disequilibrium were drawn using the R package snp.plotter [39].

## Supporting Information

Figure S1 Quantile-quantile plot.
(0.65 MB EPS)

Table S1 Complete results for all 152 stature loci.
(0.28 MB XLS)

Table S2 Summary of genome-wide imputation.
(0.02 MB XLS)
